# Predicting dementia in primary care patients with a cardiovascular health metric: a prospective population-based study

**DOI:** 10.1186/s12883-016-0646-8

**Published:** 2016-07-26

**Authors:** Johannes Baltasar Hessler, Karl-Heinz Ander, Monika Brönner, Thorleif Etgen, Hans Förstl, Holger Poppert, Dirk Sander, Horst Bickel

**Affiliations:** 1 Department of Psychiatry and Psychotherapy, Klinikum rechts der Isar, Technical University of Munich, Ismaninger Strasse 22, D-81675 Munich, Germany; 2 INVADE Study Group, Karl-Böhm-Strasse 32, D-85598 Baldham, Germany; 3 Department of Neurology, kbo-Inn-Salzach-Klinikum, Gabersee 7, D-83512 Wasserburg am Inn, Germany; 4 Department of Neurology, Klinikum rechts der Isar, Technical University of Munich, Ismaninger Strasse 22, D-81675 Munich, Germany; 5 Department of Neurology, Benedictus Krankenhaus Tutzing, Bahnhofstrasse 5, D-82327 Tutzing, Germany

**Keywords:** Dementia prevention, Modifiable risk factors, Cardiovascular disease, Life’s simple 7

## Abstract

**Background:**

Improving cardiovascular health possibly decreases the risk of dementia. Primary care practices offer a suitable setting for monitoring and controlling cardiovascular risk factors in the older population. The purpose of the study is to examine the association of a cardiovascular health metric including six behaviors and blood parameters with the risk of dementia in primary care patients.

**Methods:**

Participants (*N* = 3547) were insurants aged ≥55 of the largest German statutory health insurance company, who were enrolled in a six-year prospective population-based study. Smoking, physical activity, body mass index, blood pressure, total cholesterol, and fasting glucose were assessed by general practitioners at routine examinations. Using recommended cut-offs for each factor, the patients’ cardiovascular health was classified as ideal, moderate, or poor. Behaviors and blood parameters sub-scores, as well as a total score, were calculated. Dementia diagnoses were retrieved from health insurance claims data. Results are presented as hazard ratios (HRs) and 95 % confidence intervals (95 % CIs).

**Results:**

Over the course of the study 296 new cases of dementia occurred. Adjusted for age, sex, and education, current smoking (HR = 1.77, 95 % CI 1.09–2.85), moderate (1.38, 1.05–1.81) or poor (1.81, 1.32–2.47) levels of physical activity, and poor fasting glucose levels (1.43, 1.02–2.02) were associated with an increased risk of dementia. Body mass index, blood pressure, and cholesterol were not associated with dementia. Separate summary scores for behaviors and blood values, as well as a total score showed no association with dementia. Sensitivity analyses with differently defined endpoints led to similar results.

**Conclusions:**

Due to complex relationships of body-mass index and blood pressure with dementia individual components cancelled each other out and rendered the sum-scores meaningless for the prediction of dementia.

## Background

The number of people with dementia is expected to increase due to the aging of the global population [1]. Along with this increase come high and rising costs for care, rendering dementia a worldwide public health problem [2]. In the absence of pharmacological treatment that goes beyond symptom delay or reduction, the study of modifiable risk factors for dementia has high priority [3]. Converging evidence points to a central role of cardiovascular risk factors such as physical inactivity, smoking, diabetes mellitus, midlife hypertension, midlife obesity, and hyperlipidemia in late-life cognitive decline [4].

Several studies suggested that substantial fractions of dementia cases could be prevented if modifiable cardiovascular risk factors were targeted by intervention [5–7]. Evidence-based prevention programs, however, have only recently been developed [8] and are far from implementation on the population level [3]. Primary care practices might offer a suitable setting for delivering preventive measures, as they provide the facilities to assess and control cardiovascular risk factors for dementia in the older general population. In this context it would be desirable to have a tool for the assessment of cardiovascular health (CVH) that can be easily employed by general practitioners (GPs) and that facilitates the communication between patient and physician.

A range of CVH-metrics have been employed to predict cognitive decline and dementia [9]. Among the more recent is the Life’s Simple 7 metric that was developed by the American Heart Association (AHA) [10]. The Life’s Simple 7 assess CVH based on seven parameters: Smoking, body mass index (BMI), physical activity, dietary habits, total cholesterol, blood pressure, and fasting glucose. Cut-offs are applied to each parameter to categorize the patients CVH-status as poor, moderate, or ideal. Based on these classifications two sub-score for health behaviors and blood parameters, as well as a total score can be calculated. The simple three-step approach has illustrative value and has been combined with a traffic-light system in primary care to improve patient-physician communication and track changes in CVH as an effort to prevent stroke in primary care [11].

So far, however, only few studies investigated the relationship between the Life’s Simple 7 and cognition. Low scores indicating poor CVH were associated with decreased performance on measures of cognitive functioning in a cross-sectional investigation [12] and predicted incident cognitive impairment in previously unimpaired and stroke-free persons [13]. Furthermore, high scores indicating good CVH in young adulthood were related to better cognitive functioning in mid-life [14]. To the best of our knowledge, the Life’s Simple 7 have not been used to predict dementia diagnosed according to clinical criteria. Also, no study so far examined whether a CVH-metric can be employed to assess dementia risk in primary care, where most older people receive their health care and where dementia prevention programs are likely to be implemented.

As age is the most important predictor for dementia, CVH-metrics need to provide additional information to be useful. Yet, this characteristic cannot always be assumed. A commonly employed CVH-metric, for example, was found to lose all predictive validity for dementia death when age was included in the model [15].

The aim of our study was to determine the potential of a CVH-metric to identify individuals with increased cardiovascular risk of dementia at routine primary care visits. Since the data of the present study were collected before the Life’s Simple 7 was introduced, it was not possible to fully adhere to the criteria proposed by the AHA. Instead, the association between scores on a CVH-metric whose construction was based on the Life’s Simple 7 and incident dementia was examined in a large sample of older primary care patients. In particular, it was investigated whether behavioral variables, blood parameters, or a combination best captured the cardiovascular risk for dementia and whether possible associations are independent of age, sex, and educational level.

## Methods

### Participants

The present study was conducted as part of the INVADE-trial (Intervention Project on Cerebrovascular Disease and Dementia in the District of Ebersberg), a prospective and population-based cohort study in a geographically defined area in southern Germany [16]. Participants were identified from the database of the statutory health insurance company AOK (Allgemeine Ortskrankenkasse). In Germany, membership in a health insurance is mandatory and the AOK holds the largest market share, representing around 40 % of the total population. In 2001, 11,317 insurants met the inclusion criteria of being older than 54 years, as well as living in the district of Ebersberg, and were invited to participate. Three-thousand nine-hundred and eight participants enrolled between 2001 and 2003. The observation period ended in 2008.

### Procedure

The ethics committee of the Faculty of Medicine at the Technische Universität München approved the study protocol and all participants signed informed consent.

The participants were examined by their GPs. The GPs reported the patients’ previous and current diagnoses, current medication, alcohol consumption, physical activity, BMI, impairment of activities of daily living (Rankin Scale [17]), ankle-to-brachial index, cognitive status (6-Item Cognitive Impairment Test [18, 19]), and conducted an electrocardiogram. Blood pressure was measured in a supine position twice with an interval of five minutes and a mean value was calculated. The GPs also took fasting blood samples that were analyzed in a central laboratory with regard to total cholesterol, low- and high-density lipoprotein cholesterol, triglycerides, serum glucose, glycosylated hemoglobin A_1c_, creatinine, homocysteine, and high-sensitivity C-reactive protein, and measured ankle-to-brachial index. The participants filled in questionnaires about sociodemographic data, depressive symptoms (Geriatric Depression Scale [20]), use of medical services, memory complaints, and subjective health. The INVADE-trial and the baseline examination are described in further detail elsewhere [16].

### Cardiovascular health metric and dementia diagnoses

The CVH-metric employed in the present study is based on the Life’s Simple 7, as they include both behavioral and blood parameters and propose a comprehensible and communicable scoring system. Since the data of our study were collected before the Life’s Simple 7 was introduced, it was not possible to completely adhere to the original criteria. The Life’s Simple 7 is, hence, not directly examined in the present study. Four adjustments had to be made to fit the metric to the data at hand: (1) No information about time since smoking cessation were available; (2) dietary habits were not recorded during the INVADE trial and therefore dropped from the metric; (3) physical activity was assessed as number of vigorous activities per week, not minutes per week; and (4) antidiabetic medication was not recorded in sufficient detail to be considered. Table 1 depicts the CVH-metric employed to predict dementia.

**Table 1:** Components and scoring of the cardiovascular health metric

Component	Scoring		
	Ideal (2 points)	Moderate (1 point)	Poor (0 points)
Smoking^a^	Never	Quitter	Current
Physical activity^a^	≥3 times/week	1–2 times/week	inactive
Body mass index^a^	<25 kg/m^2^	25–29 kg/m^2^	≥30 kg/m^2^
Blood pressure^b^	SBP < 120 and DBP < 80 mmHg untreated	SBP 120–139 or DBP 80–89 mmHg or ideal but treated	SBP ≥ 140 or DBP ≥ 90 mmHg
Total cholesterol^b^	<200 mg/dl untreated	200–239 mg/dl or ideal but treated	≥240 mg/dl
Fasting glucose^b^	<100 mg/dl	100–125 mg/dl	≥126 mg/dl

^a^Components of the health behaviors index. ^b^Components of the blood parameters index. *DBP* diastolic blood pressure. *SBP* systolic blood pressure

Using the variables and cut-offs from Table 1 three indices were calculated by adding up individual component scores. (1) A health behaviors index, including smoking, physical activity, and BMI (range 0–6). (2) A blood parameters index, including blood pressure, total cholesterol, and fasting glucose (range 0–6). (3) A sum score of all individual component scores (range 0–12). All three indices were recoded to ensure sufficient group sizes for meaningful comparisons.

Dementia diagnoses over the course of the study were retrieved from health insurance claims data. This method has been shown to produce sufficiently valid case classifications [21]. All diagnoses listed in the ICD-10 under F00 – F03 and G30 – G31 were included. To increase the validity of the diagnoses, the incident dementia cases were narrowed to participants who received respective diagnoses in at least two (not necessarily consecutive) billing quarters of the statutory health insurance or in both in- and outpatient settings, as has been previously done in a large-scale analysis of claims data from the same insurance company [22]. Participants who received a diagnosis only once were excluded from the analysis.

### Statistical analyses

Cox proportional hazards regressions were employed to estimate the risk of dementia associated with the individual components and combined scores of the CVH-metric. All variables were treated as categorical and for each variable an individual model was built. Both time variable and clinical endpoint were the same for all analyses. For incident dementia cases, the time variable was defined by the time in month between the baseline examination and the date of the first diagnosis according to health insurance claims data. Cases without incident dementia were censored at the end of observation (change of insurance, study end, or death). All Cox proportional hazards regression models were then performed again with adjusting each model for age in years, sex, and education (no formal degree; primary compulsory education, ≈ 8 years; higher degrees, ≥ 10 years).

Sensitivity analyses with varying definitions of the clinical endpoint were calculated. First, Cox proportional hazards regression models were built as described above. Instead of the strict outcome variable (dementia diagnoses in two billing quarters or settings), a more liberally defined clinical endpoint was used (at least one billing quarter or setting). Second, the same analyses were performed with those cases that received only one diagnosis (one quarter or setting) counted as non-dementia cases instead of being excluded from the analysis.

Results of the Cox proportional hazards regression models are reported as hazard ratios and 95 % confidence intervals. Data analysis was performed with SPSS 22 for Microsoft Windows (IBM, Armonk, New York).

## Results

**Fig. 1:**
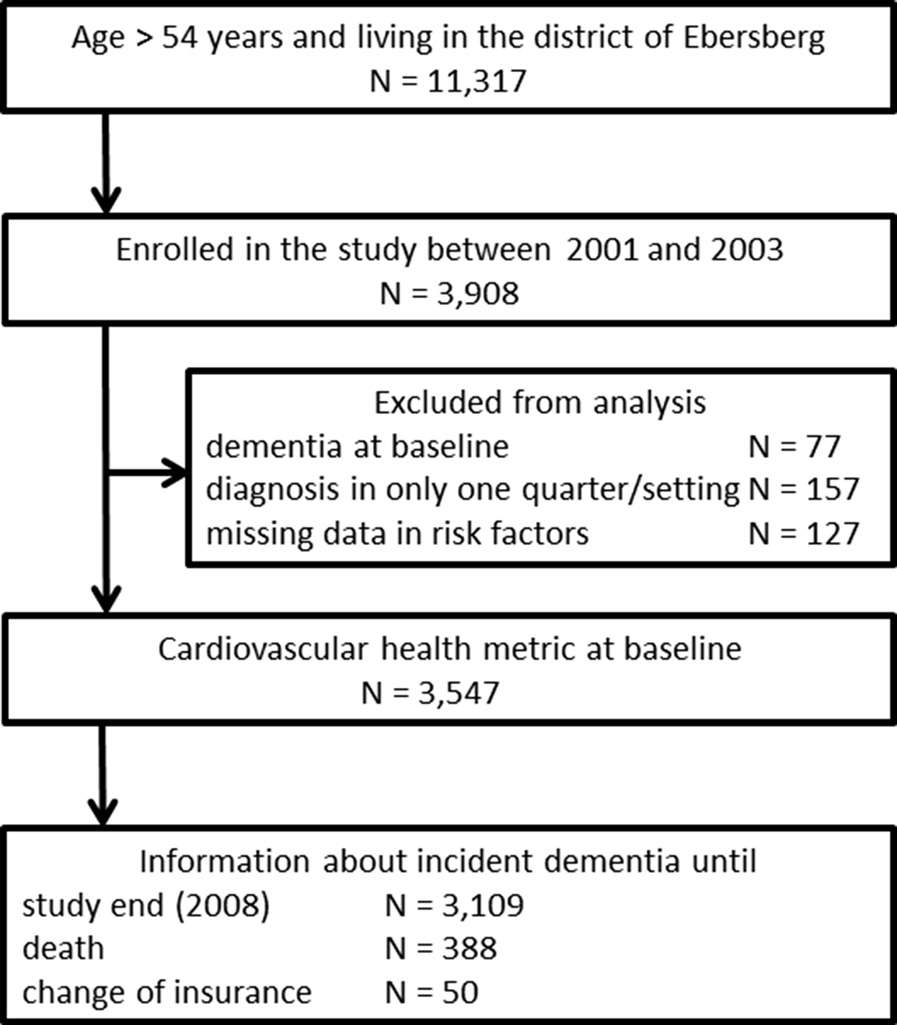
Participant flow chart

At baseline, 3908 participants were examined. Three-hundred sixty-one participants were excluded from the analysis (Fig. 1). The baseline characteristics of the included 3547 persons are displayed in Table 2. The median observation time was 6.7 years. In total, 296 (8.3 %) new cases of dementia occurred over the course of the study. Most diagnoses (*N* = 170, 57.4 %) pertained to “dementia not otherwise specified” (ICD-10 F03) so that no sub-analysis for different types of dementia was performed.

**Table 2:** Sample characteristics at baseline

Characteristics	Examined at baseline *N* = 3547
Age; M (SD)	67.28 (7.57)
Female; N (%)	2101 (59.2)
Education; N (%)	
No formal degree	166 (4.7)
Primary compulsory	3007 (84.7)
Higher degrees	375 (10.6)
Observation time in years; median	6.7
Incident dementia; N (%)	296 (8.3)

Over the course of the study, 388 patients were lost due to death and 50 due to a change of the insurance company. For all other cases information about incident dementia was available until the study end. That is, for merely 50 participants there is only limited information about incident dementia (from start of the observation until change of insurance).

The proportional hazards assumption was checked by visual inspection of Kaplan-Meier estimator plots and found to be valid. Table 3 displays the results of both the unadjusted and adjusted Cox proportional hazards regression models for the individual components. In the unadjusted model, moderate and poor levels of physical activity, as well as poor levels of fasting glucose were statistically significantly associated with an increased risk of dementia. Poor levels of BMI, however, were associated with a decreased risk. Adjusting for age, sex, and education revealed significant associations of physical activity and fasting glucose with dementia that were similar to the unadjusted analyses, yet slightly decreased in strength. Poor smoking behavior (i.e., current smoking) was now associated with a significantly increased risk of dementia, possibly due to the fact that most smokers in the sample were of younger age. Poor BMI status was not anymore a significant predictor, indicating a stronger relationship of BMI with demographic variables than with dementia. All other models remained unchanged by the adjustment.

**Table 3:** Cardiovascular health components, group sizes, and dementia risk. Results of the Cox proportional hazards regressions

Cardiovascular health component scores^a^	Dementia/total *N*	Unadjusted models HR (95 % CI)	Adjusted models^b^ HR (95 % CI)
Smoking			
2 (ideal)	214/2366	Reference	Reference
1 (moderate)	61/821	0.84 (0.63–1.12)	1.15 (0.83–1.61)
0 (poor)	21/360	0.66 (0.42–1.03)	**1.77 (1.09–2.85)**
Physical activity			
2 (ideal)	89/1672	Reference	Reference
1 (moderate)	126/1384	**1.80 (1.37–2.36)**	**1.38 (1.05–1.81)**
0 (poor)	81/491	**3.53 (2.61–4.76)**	**1.81 (1.32–2.47)**
Body mass index			
2 (ideal)	99/980	Reference	Reference
1 (moderate)	141/1645	0.82 (0.64–1.07)	0.98 (0.76–1.28)
0 (poor)	56/922	**0.58 (0.42–0.81)**	0.77 (0.55–1.07)
Blood pressure			
2 (ideal)	9/182	Reference	Reference
1 (moderate)	122/1460	1.76 (0.90–3.47)	0.90 (0.46–1.79)
0 (poor)	165/1905	1.83 (0.93–3.57)	0.74 (0.37–1.45)
Total cholesterol			
2 (ideal)	88/911	Reference	Reference
1 (moderate)	130/1613	0.82 (0.63–1.08)	0.81 (0.62–1.06)
0 (poor)	78/1023	0.78 (0.58–1.06)	0.86 (0.63–1.16)
Fasting glucose			
2 (ideal)	197/2511	Reference	Reference
1 (moderate)	59/689	1.10 (0.82–1.47)	0.84 (0.63–1.13)
0 (poor)	40/347	**1.57 (1.12–2.20)**	**1.43 (1.02–2.02)**

**Boldface** indicates statistical significance at *p* < 0.05. ^a^Higher scores indicate better cardiovascular health. ^b^Adjusted for age, sex, and education. *HR* hazard ratio. *95 % CI* 95 % confidence interval

Table 4 displays the unadjusted and adjusted Cox proportional hazards regression models for the behavioral index, the blood parameters index, and the total score. None of the scores was able to predict dementia, neither with nor without adjustment. Sensitivity analyses with varying definitions of the clinical endpoint generally confirmed the previously established associations and, thereby, support the validity of the original endpoint. Using the more liberal endpoint (i.e., diagnosis only in at least one billing quarter or setting) most associations between CVH-variables and dementia remained unchanged in strength and direction. Counting the 157 cases with only one diagnosis as non-dementia cases most associations decreased in strength but retained their direction.

**Table 4:** Cardiovascular health sum scores, group sizes, and dementia risk. Results of the Cox proportional hazards regressions

Cardiovascular health scores^a^	Dementia/total *N*	Unadjusted models HR (95 % CI)	Adjusted models^b^ HR (95 % CI)
Health behaviors^c^			
4–6	192/2315	Reference	Reference
3	62/719	1.06 (0.80–1.41)	0.98 (0.73–1.31)
0–2	42/513	1.03 (0.74–1.44)	1.28 (0.91–1.80)
Blood parameters^d^			
4–6	104/1299	Reference	Reference
3	92/1174	0.98 (0.74–1.30)	0.79 (0.60–1.05)
0–2	100/1074	1.20 (0.91–1.57)	0.95 (0.72–1.25)
Total score^e^			
9–12	52/752	Reference	Reference
5–8	212/2458	1.28 (0.95–1.74)	0.98 (0.72–1.33)
0–4	32/337	1.47 (0.94–2.28)	1.41 (0.91–2.20)

^a^Higher scores indicate better cardiovascular health. ^b^Adjusted for age, sex, and education. ^c^Sum of smoking, physical activity, and body mass index. ^d^Sum of blood pressure, total cholesterol, and fasting glucose.^e^Sum of smoking, physical activity, body mass index, blood pressure, total cholesterol, and fasting glucose. *HR* hazard ratio. *95 % CI* 95 % confidence interval

## Discussion

The present study investigated whether a CVH-metric combining health behaviors and blood parameters could be used to identify primary care patients at cardiovascular risk of dementia and, hence, in need of medical intervention. In sum, the results do not support the suitability of the employed CVH-metric for a valid assessment of the cardiovascular risk of dementia in older primary care patients.

Employing a CVH-metric that combines several factors to assess dementia risk is only justified if the sum scores carry predictive value that goes beyond the information gained from the individual components. Also, the metric should add information about dementia risk independently of age and other demographic variables. Both assumptions were not found to hold true for the CVH-metric employed in the study at hand. Only the individual components smoking, physical activity, and fasting glucose were significant predictors. Sub-scores for health behaviors and blood parameters, as well as a total score were not able to capture the cardiovascular risk of dementia. With regard to the research question it can be concluded that the employed metric is not suited to assess the risk of dementia attributable to cardiovascular disease in a demographically heterogeneous sample of primary care patients.

Inspecting the demographically adjusted associations between the individual CVH-components and dementia reveals the problems underlying the sum-scores. Current smoking, sedentary behavior, and impaired fasting glucose were scored as reflecting poor CVH and showed the expected relationship with an increased dementia risk. Contrary, poor levels of BMI, blood pressure, and total cholesterol were actually related to a (statistically non-significant) decreased risk of dementia. By adding the individual components into sum scores, they likely cancelled each other out and rendered the derived scores useless. That is, the sum scores actually masked the valuable predictive information that is included in the single components.

The problems encountered with the CVH-metric as tool to assess the cardiovascular risk of dementia reflect emerging evidence that suggests non-linear relationships of blood pressure and BMI with dementia where age acts as a moderating factor. While high blood pressure at mid-life seems to increase the risk of dementia, the association appears to reverse at late-life, when higher levels are associated with a decreased risk [23]. The results of the study at hand support this notion. Similarly, overweight and obesity at mid-life are assumed to increase the risk of dementia [24], while a higher BMI in late-life seems to be associated with a decreased risk [25, 26]. In addition, the relationship of hypercholesterolemia with dementia is somewhat ambiguous and requires further investigation [27, 28], yet, the condition would be expected to relate to an increased risk [29]. In the study at hand, no relationship was found. The ordinal three-step conceptualization of the CVH-metric (poor, moderate, ideal) appears unsuited to capture these complex associations of blood pressure, BMI, and cholesterol with dementia. As a consequence, a CVH-metric that might be well suited to predict, for example, mortality [30] is likely not applicable for the assessment of the cardiovascular risk of dementia.

Reverse causality also needs to be considered when interpreting the results. It is possible that preclinical dementia actually causes weight loss and a decrease in blood pressure, which can be observed years before a clinical diagnosis of dementia is made. In this case, hypotension and underweight would rather constitute early consequences of incipient dementia than risk factors. These potential associations might account for the above described interaction of BMI and blood pressure with age in the pathogenesis of dementia. When administered at only one point in time at higher age, CVH-metrics would not be able to capture these decade-long processes and red-flag potential underlying cognitive decline. Instead, CVH-metrics needed to be administered repeatedly starting at mid-life in order to track, for example, progressive weight loss that might indicate incipient dementia.

The CVH-metric employed in the present study slightly deviates from the Life’s Simple 7 with regard to the employed variables and cut-offs. Consequently, the findings may not fully pertain to the Life’s Simple 7. The most important difference was that diet was not included. Even though a healthy diet seems to be associated with a decreased risk of dementia [31, 32], previous studies found no or only weak associations between diet and cognition in the context of the Life’s Simple 7 [12–14]. Therefore, it seems unlikely that the omission of diet significantly decreased the predictive validity of the CVH-metric. Also, physical activity was defined as vigorous activity, hence, not taking into account light or moderate activity.

Previous studies [12–14] reported stronger associations between the Life’s Simple 7 total sum score and cognitive impairment than the association between the CVH-metric and dementia. Next to differences in the components of the metrics, these studies employed measures of specific cognitive functions as outcome variables. Contrary, in the present study a clinical diagnosis of dementia was used. It is possible that others studies found associations between poor general CVH and more subtle cognitive changes that would not suffice to justify a diagnosis of dementia.

If the goal is to develop a CVH-metric with high predictive validity, it should only include components that show unambiguous associations with dementia. Smoking [33, 34], sedentary behavior [35, 36], and hyperglycemia [37], are well-known to substantially contribute to late-life cognitive decline. Focusing on these variables might offer a starting point for the assessment and reduction of the cardiovascular risk of dementia. Metrics could also be used to monitor improvements in CVH. In the Life’s Simple 7, improved CVH would be reflected in increasing scores. It remains to be investigated, however, whether improving scores actually translate into a decreasing dementia risk.

The strengths of the present study include that CVH was assessed at routine primary care examinations by the participants’ usual GPs. The results, therefore, reflect current possibilities to monitor and control cardiovascular risk factors in the health care system. Only for a very small number of participants there was incomplete information about incident dementia available. This fact reduces the possibility of bias due to non-random drop-out. These diagnoses were made in in- and outpatient settings according to usual clinical practice and, thereby, increase the study’s relevance and validity in the health care system. Some limitations had to be accepted. The use of ICD-codes as clinical endpoints may bear the risk of falsely classified cases. In order to reduce the possibility of overstated results due to false positives and increase the validity of the endpoint, more conservative criteria were applied to the outcome variable and sensitivity analyses were conducted. False negative cases also might have been an issue, assuming that dementia is underdetected in community-dwelling persons. However, dementia case definitions based on health insurance claims data have been shown to be sufficiently valid [21, 22]. Two-thirds of the eligible patients did not enroll in INVADE. Given that the study was aimed at the identification and treatment of cardiovascular disease, it is possible that more health-conscious people decided to participate. Potentially, these persons showed better CVH than the general older population, which might somewhat reduce the generalizability of the study’s findings.

## Conclusion

The study at hand was the first to examine the suitability of a CVH-metric to assess the cardiovascular risk of dementia for older persons at routine primary care examinations. The metric’s sum-scores were not associated with dementia risk and, therefore, do not seem to be suited for that purpose. Due to complex relationships of BMI and blood pressure with dementia, individual components cancel each other out and render the sub- and sum-scores useless. CVH-metrics that are suitable for other purposes cannot be readily applied for assessing the risk of dementia due to cardiovascular disease on the population level.

## Abbreviations

AHA, American Heart Association; AOK, Allgemeine Ortskrankenkasse; BMI, body mass index; CI, confidence interval; CVH, cardiovascular health; DBP, diastolic blood pressure; GP, general practitioner; HR, hazard ratio; IBM, International Business Machines Corporation; ICD-10, International Classification of Diseases and Related Health Problems 10th revision; INVADE, Intervention Project on Cerebrovascular Disease and Dementia in the District of Ebersberg; SBP, systolic blood pressure; SPSS 22, Statistical Package for the Social Sciences version 22
